# Quaternarization and polymerization of 2-chloroethyl maleate derivative of epoxidized soybean oil

**DOI:** 10.55730/1300-0527.3503

**Published:** 2022-10-04

**Authors:** Demet GÜRBÜZ

**Affiliations:** Department of Chemistry, Faculty of Engineering, İstanbul University-Cerrahpaşa, İstanbul, Turkey

**Keywords:** Epoxidized soybean oil, maleate esters, quaternary ammonium salts, renewable resources, plant oil triglycerides

## Abstract

As a potent antibacterial, antifungal or antiviral material, synthesis of quaternarized epoxidized soybean oil was demonstrated in this work. The first step of the synthesis is the reaction of epoxidized soybean oil (ESO) with 2-chloroethyl maleic acid (Cl-MA-ESO). The last step is quaternarization of this condensate with triethylamine (Q-MA-ESO). All the synthesized monomers were characterized by FTIR and ^1^H NMR techniques. Homopolymer of the quaternarized derivative (Q-MA-ESO) was not obtained. However, styrene copolymers were obtained and characterized. Thermal properties of the copolymers were determined by TGA and DSC techniques. Additionally, swelling properties of the materials were also evaluated. The highest swelling ratio was observed in toluene.

## 1. Introduction

Throughout history, human being has seen the world as an inexhaustible resource. Especially following the industrial revolution, mankind has turned to overconsumption and all resources have nearly come to an end for the sake of development, until the balance of the world was disturbed. After some serious environmental warnings such as climate change, various environmental problems and diseases, new economic models were developed. The most important is sustainable development model. For sustainable development, it is vital to find cheap and appropriate materials from renewable resources. There are many types of renewable material that can be obtained from natural resources. Among them, plant oil triglycerides, which have a complex nature, are taking special attentions. They are inexpensive and readily available. They could also be used to produce many valuable chemicals and polymers [1–4]. Because of the reactive sides that triglycerides possess, one can easily modify plant oil triglycerides. The main reactive positions of plant oil triglycerides are ester groups, α carbon to ester groups, double bonds and allylic positions [5–8]. Among those reactive parts, manipulations of double bonds are much easier than modification of other parts.

Among the plant oil triglycerides, soybean oil is the second most produced one. Reaction of soybean oil with peroxy acids yields epoxidized derivative of soybean oil (ESO). Epoxidized soybean oil is an important intermediate because it can be used as an epoxy resin. ESO has an average of 4.5 epoxy group and it is a valuable renewable material for many industrial applications [9–21]. Many types of complex monomers can be synthesized via modification of ESO. Epoxy rings of the ESO give addition reactions with some nucleophiles and electrophiles. Amines, phenols and carboxylic acids can be used as a nucleophile. The final materials are hydroxyl group-containing compounds.

There are many types of addition reaction of ESO and the products of those reactions in the literature. However, there is not enough information about cationic group-containing ESO-based materials. The most known cationic compounds are quaternary ammonium salts. They can be used as an antibacterial [22], antiviral [23], and antifungal [24] materials. Their activation mechanism is via neutralizing the charge on the living cell or blocking the active channel on the cell membrane.

**Figure 1:**
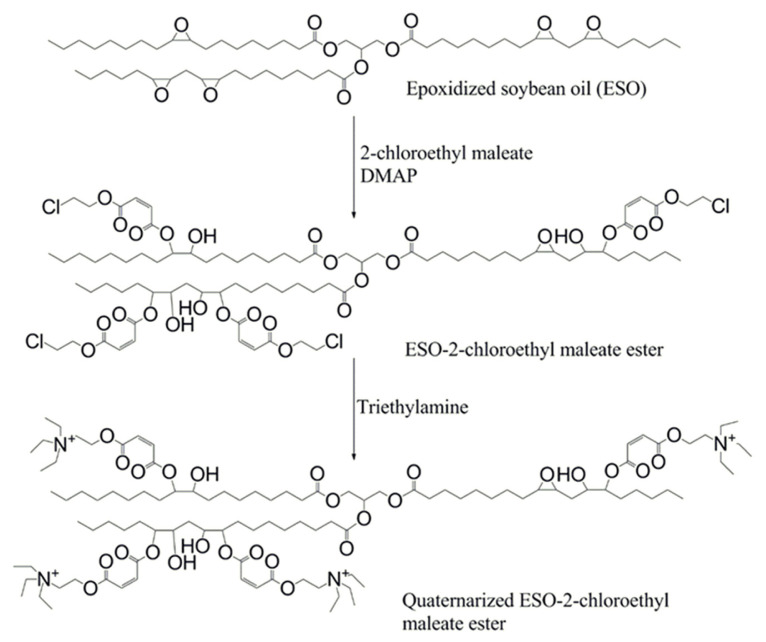
Schematic representation of the synthesis of monomer.

The synthesis and polymerization of quaternarized derivative of 2-chloroethyl maleate condensate of epoxidized soybean oil (Q-MA-ESO) was carried out in this study. The synthesis of the monomer was achieved in two-step. Condensation reaction of 2-chloroethyl maleate and ESO was completed at the first step and then, this monomer was quaternarized with triethylamine (Figure 1). Copolymers of Q-MA-ESO were also obtained. To the best of my knowledge, this monomer is the first example in the literature.

## 2. Materials and methods

### 2.1. Chemistry

Epoxidized Soybean Oil (ESO) was obtained from Sankim (İstanbul, Turkey), maleic anhydride (MA), 2-chloroethanol (CE), 4-(dimethylamino)pyridine (DMAP), zinc chloride, hydroquinone, styrene (ST), sodium iodide, tetrahydrofuran (THF), triethylamine (TEA) were purchased from Merck (Darmstadt, Germany). All of these chemicals were used without any further purification. FTIR spectra of the compounds and copolymers were performed by Bruker Optics Vertex 70 spectrometer using ATR (attenuated total reflection) techniques from 4000 cm^−1^ to 400 cm^−1^. ^1^H NMR spectra were acquired by a Varian Unity Inova 500 NMR spectrometer in CDCl_3_. Thermal stabilities of the material were found by Shimadzu DTG 60 model TGA instrument with a 10 °C/min heating rate under nitrogen atmosphere. Differential scanning calorimetric (DSC) analyses of the copolymers were performed with a heat-flux type DSC instrument (SII Nanotechnology, ExStar 6200).

### 2.2. Synthesis of 2-chloroethyl maleate

In a 250-mL flask, 19.6 g of maleic anhydride (0.2 mol) and 16.1 g of 2-chloroethanol (0.2 mol) were dissolved in 100 mL of tetrahydrofuran and then, 0.1 g of zinc chloride was added to this mixture to initiate the reaction. The mixture was stirred and refluxed for 8 h. After completion of reaction, the tetrahydrofuran was removed by using a rotary evaporator.

### 2.3. Synthesis of 2-chloroethyl maleate condensate of ESO

For synthesis of 2-chloroethyl maleate condensate of ESO (Cl-MA-ESO), 9.5 g of ESO (0.01 mol) was reacted with 7.15 g of 2-chloroethyl maleate (0.04 mol) in the presence of 0.05 g of DMAP as the catalyst for ring-opening of ESO in a two-necked round-bottomed flask fitted with a reflux condenser under stirring. The reaction mixture was heated for 6 h at 90 °C under nitrogen atmosphere. The resultant mixture was used without any further purification.

### 2.4. Quaternarization of 2-chloroethyl maleate condensate of ESO

A solution of 10 g of Cl-MA-ESO, 10 g of TEA and 50 mL of THF in a two-necked round**-**bottomed flask was prepared. Then, 0.05 g of hydroquinone and 0.01 g sodium iodide were added to this mixture. The reaction mixture was heated under nitrogen atmosphere for 12 h at 90 °C. After completion of reaction, the tetrahydrofuran and excess TEA were removed by using a rotary evaporator.

### 2.5. Polymerization of quaternarized 2-chloroethyl maleate condensate of ESO

Nitrogen gas was purged into the monomer mixture that contains 1% of benzoyl peroxide for 10 min. Then, the mixture was kept in an oven at 80 °C for 3 h followed by further curing at 85 °C for 12 h.

## 3. Results

### 3.1. Characterization of monomer

**Figure 2:**
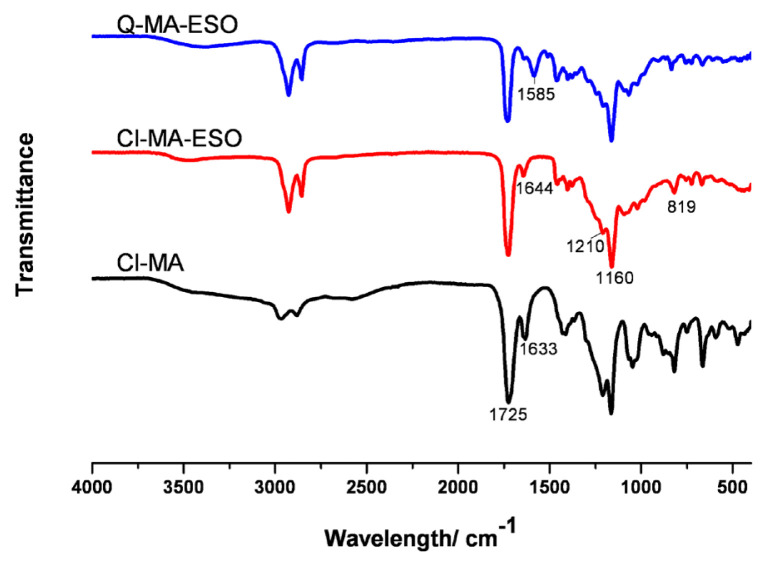
FTIR spectra of Cl-MA, Cl-MA-ESO, and Q-MA-ESO.

Characterization of the monomer was examined by using FTIR and NMR techniques. FTIR spectra of the synthesized compounds are shown in Figure 2. The peak of ester group is observed at 1741 cm^−1^ as a sharp and strong peak. Epoxy peaks appear at 830 cm^−1^ as a weak peak. As a result of reaction of 2-chloroethanol with maleic anhydride, an ester peak appeared at 1725 cm^−1^ as a strong peak. The peak at 1633 cm^−1^ is assigned to double bonds of maleate group. A broad peak between 2500 cm^−1^ and 3500 cm^−1^ was determined, which indicated the presence of free carboxyl group. As a result of reaction of 2-chloroethyl maleate with ESO, the peaks of free carboxyl groups disappeared. The peak at 1644 cm^−1^ belongs to double bond of maleate groups instead of 1633 cm^−1^. The peaks at 1210 and 1160 cm^−1^ indicates C-O bending vibration. Additionally, the peak observed at 819 cm^−1^ shows the presence of C-Cl bond. After the completion of the quaternarization, that peak disappeared and an additional peak at 1585 cm^−1^ is observed, which shows the presence of C-N bond.

**Figure 3:**
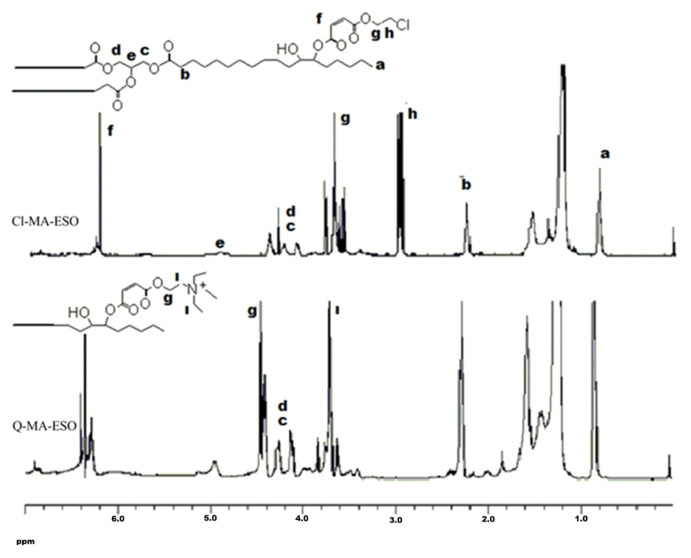
^1^H NMR spectra of Cl-MA-ESO and Q-MA-ESO.

Another method to investigate the structure of a molecule is ^1^H NMR spectrum. The ^1^H NMR spectrum of the synthesized compounds is shown in Figure 3. According to the literature, epoxy peaks appear at 3.2 ppm. The peaks that are observed at 4.1, 4.3, and 5.1 ppm belong to hydrogen of glycerol moiety. The peak at 0.9 ppm is ascribed to methyl groups of fatty acids. Hydrogens of α-methylene group to carbonyl appear at 2.25 ppm and β methylene hydrogens to carbonyl appear at 1.6 ppm [25]. When ESO was reacted with 2-chloroethyl maleate, it was observed that the peaks at 3.2 ppm disappeared. This observation indicated that all epoxy groups were reacted with maleate ester. Additionally, new peaks appeared at 3.0 and 3.65 ppm, which indicates the presence of α-methylene hydrogens to ester oxygen of maleate group and α-methylene hydrogens to chlorine atom. The peak at 6.35 ppm is ascribed to hydrogens of maleate double bond. After the quaternarization was completed, new peaks appeared at 3.65 ppm due to the presence of the hydrogens of methylene groups α to quaternary nitrogen. Additionally, the peak of the methylene hydrogens of maleate ester was shifted to 4.45 ppm.

### 3.2. Polymerization of monomer

Homopolymerization of Q-MA-ESO could not be achieved even at 3% benzoyl peroxide content. When compared to the studies in the literature, it was observed that Q-MA-ESO had a negative influence on the polymerization rate [26–27]. Thus, copolymers of the Q-MA-ESO-Styrene could be synthesized. As the Q-MA-ESO content was increased, the polymerization rate decreased. When compared with pure polystyrene, softer materials were obtained. Beyond 20% Q-MA-ESO content, it was not possible to obtain copolymers.

### 3.3. Thermal properties of polymers

**Figure 4:**
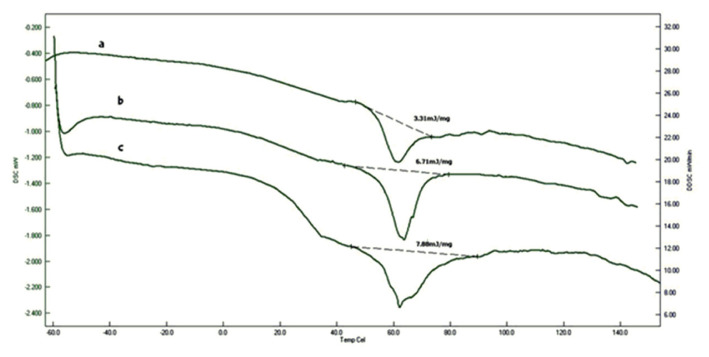
DSC thermograms of a-5%, b-10%, c-20% Q-MA-ESO containing styrene copolymers.

There are two important methods which can be used for thermal characterization of the synthesized materials. The first technique is DSC, by the aid of this technique one can readily determine Tg and melting points of the materials. DSC thermograms of the synthesized polymers are shown in Figure 4. According to this graph, all the copolymers have a Tg around −20 °C. A melting peak was observed at 62 °C. When the amount of quaternary group increased in the copolymer, Tg value did not change. However, the heat absorbed by the samples increased with the increasing percentages of quaternary groups in the copolymer structure. The highest value was determined as 7.88 J/g when 20% quaternarized monomer is introduced into copolymer. While the polymers were tried to be extracted in toluene, it could not be possible to observe any change in the weights of the samples. That would prove that all the materials were crosslinked. When Q-MA-ESO amount increased in the polymers, that would yield more amorphous structure and the melting behavior would be observed in the DSC thermograms. Although it was not produced radical polymerization technique, polymerization product of isothiocyanate derivative of soybean oil exhibited similar type of melting behavior [28]. Probably due to the presence of dangling chains of triglyceride moieties would cause this melting behavior.

**Figure 5:**
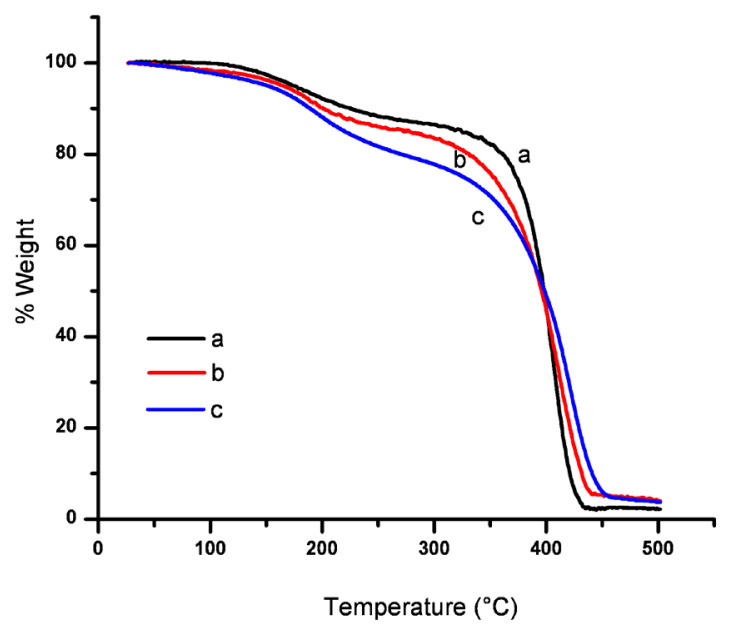
TGA thermograms of a-5%, b-10%, c-20% Q-MA-ESO containing styrene copolymers.

Another qualitative thermal characterization technique is TGA (thermogravimetric analysis). Via this technique, one can determine thermal stabilities and thermal degradation pattern of materials. TGA thermograms of the copolymer are shown in Figure 5. Among all the polymers, 5% Q-MA-ESO containing showed the highest loss of weight at 174 °C. On the other hand, the lowest weight loss appeared at 150 °C for 20% Q-MA-ESO-containing copolymer. The temperature corresponding to 50% weight loss was almost similar to that of all polymers and it was observed at 398 °C. These values can be seen at Table.

**Figure 6:**
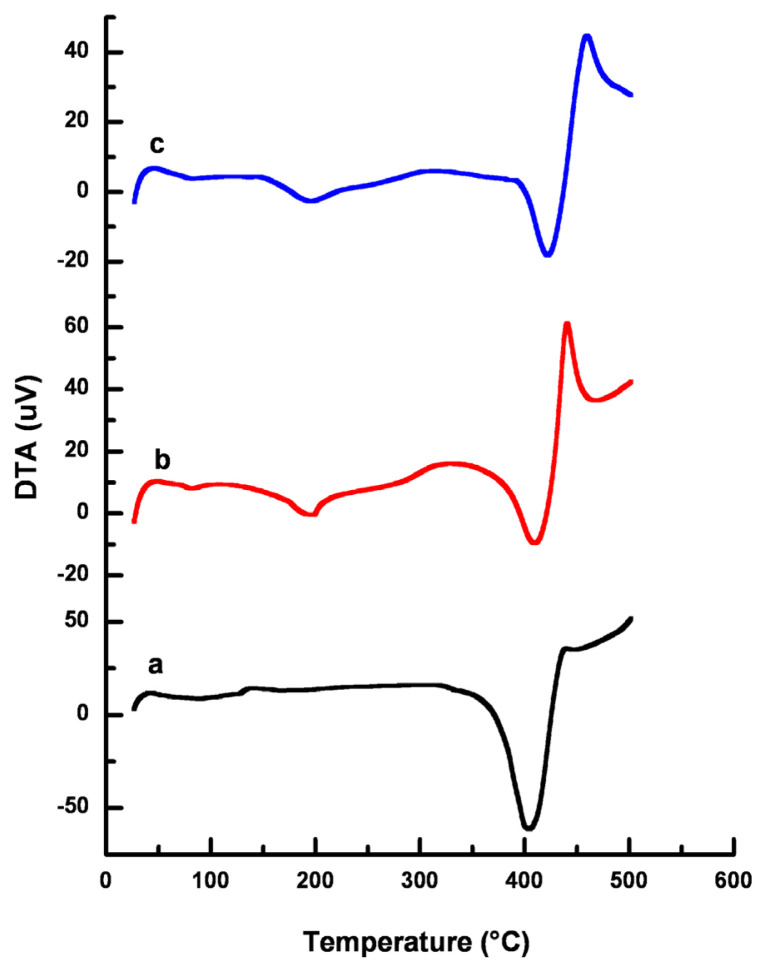
DTA curves of a-5%, b-10%, c-20% Q-MA-ESO containing styrene copolymers.

DTA values can also be used for the thermal characterization of the materials. DTA curves of the polymers synthesized are shown in Figure 6. Three main peaks were observed. Those peaks appeared at 100 °C, 195 °C, and 400 °C, respectively. Intensity of the peak at 195 °C enlarged with the increasing amount of Q-MA-ESO.

### 3.4. Swelling properties of polymers

**Figure 7:**
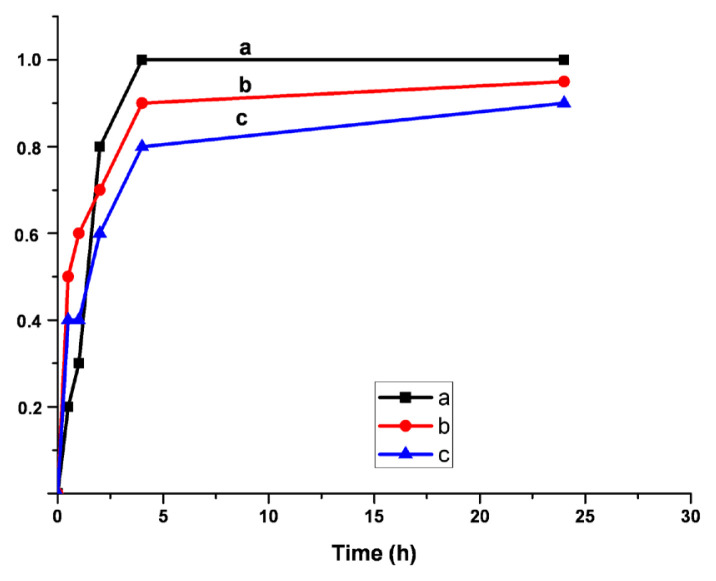
Swelling ratios a-5%, b-10%, c-20% Q-MA-ESO containing styrene copolymers in toluene.

**Figure 8:**
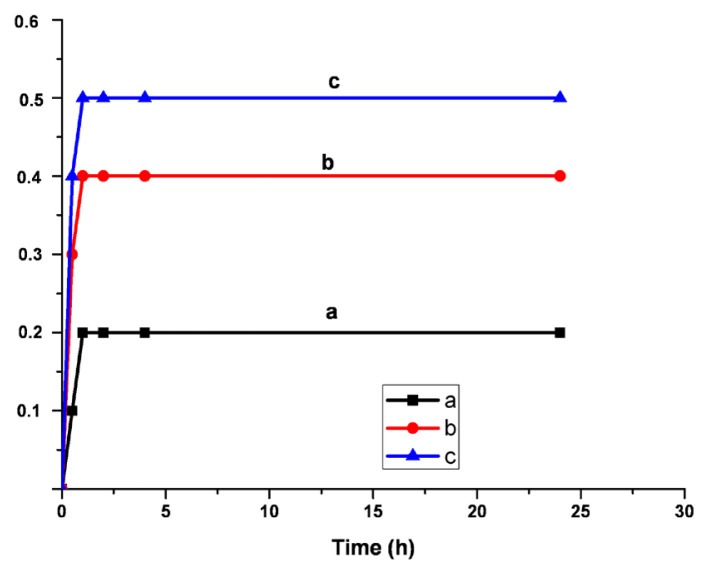
Swelling ratios a-5%, b-10%, c-20% Q-MA-ESO containing styrene copolymers in acetone.

**Figure 9:**
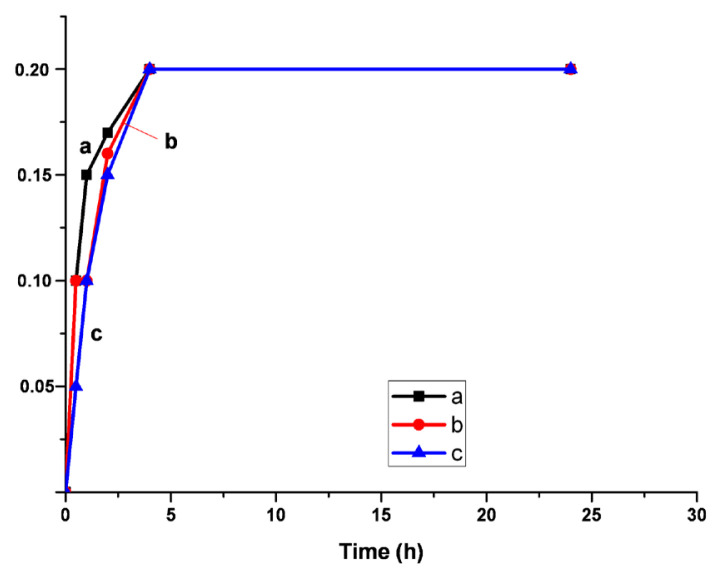
Swelling ratios a-5%, b-10%, c-20% Q-MA-ESO containing styrene copolymers in water.

The swelling behaviors of the products in toluene, acetone and water were determined using a traveling microscope. The samples were put in a closed container, and the experiment was continued until the solvent uptake ceased. The swelling ratio was expressed as follows:


$$\text{Swelling ratio}=(\text{V}/{\text{V}}_{0})={\left(\text{L}/{\text{L}}_{0}\right)}^{3},$$

where V_0_ and V are the volumes, and L_0_ and L are the length of the unswollen and swollen polymer samples, respectively. The swelling behavior of the samples are exhibited in Figures 7–9. The synthesized polymers showed the highest swelling ratios in toluene. On the other hand, the smallest ones were observed in water. In acetone, the values of swelling ratios were between in the case of water and toluene. Additionally, the shortest equilibrium time was determined in acetone. System reached the equilibrium in 1 h.

## 4. Conclusion

Unique and versatile materials were synthesized at the end of this study. By using simple and efficient techniques, a quaternary ammonium salt of 2-chloroethyl maleate ester of ESO was received. When one changes the structure of tertiary amine, different types of quaternary ammonium salts of ESO with different biological activities would be obtained. This topic would be a subject of another research that under investigation.

## Figures and Tables

**Table:** The results of thermogravimetric analysis.

Polymer composition	5% weight loss temperature	50% weight loss temperature	Char yields at 500 °C
5% Q-MA-ESO 95% styrene	174	398	2.5
10% Q-MA-ESO 90% styrene	151	396	3.9
20% Q-MA-ESO 80% styrene	150	398	4.5
